# Incorporation of Calcium Containing Mesoporous (MCM-41-Type) Particles in Electrospun PCL Fibers by Using Benign Solvents

**DOI:** 10.3390/polym9100487

**Published:** 2017-10-04

**Authors:** Liliana Liverani, Elena Boccardi, Ana Maria Beltrán, Aldo R. Boccaccini

**Affiliations:** 1 Institute of Biomaterials, Department of Materials Science and Engineering, University of Erlangen-Nuremberg, 91058 Erlangen, Germany; liliana.liverani@fau.de (L.L.); elena.boccardi@fau.de (E.B.); 2 Institute de Ciencia de Materiales de Sevilla (ICMS), CSIC-Universidad de Sevilla, Seville 41092, Spain; abeltran3@us.es; 3 Department of Engineering and Materials Science, University of Seville, Sevilla 41092, Spain

**Keywords:** electrospinning, benign solvents, mesoporous silica calcium containing MCM-41, composites, nanofibers, poly(epsilon-caprolactone)

## Abstract

The electrospinning technique is a versatile method for the production of fibrous scaffolds able to resemble the morphology of the native extra cellular matrix. In the present paper, electrospinning is used to fabricate novel SiO_2_ particles (type MCM-41) containing poly(epsilon-caprolactone) (PCL) fibers. The main aims of the present work are both the optimization of the particle synthesis and the fabrication of composite fibers, obtained using benign solvents, suitable as drug delivery systems and scaffolds for soft tissue engineering applications. The optimized synthesis and characterization of calcium-containing MCM-41 particles are reported. Homogeneous bead-free composite electrospun mats were obtained by using acetic acid and formic acid as solvents; neat PCL electrospun mats were used as control. Initially, an optimization of the electrospinning environmental parameters, like relative humidity, was performed. The obtained composite nanofibers were characterized from the morphological, chemical and mechanical points of view, the acellular bioactivity of the composite nanofibers was also investigated. Positive results were obtained in terms of mesoporous particle incorporation in the fibers and no significant differences in terms of average fiber diameter were detected between the neat and composite electrospun fibers. Even if the Ca-containing MCM-41 particles are bioactive, this property is not preserved in the composite fibers. In fact, during the bioactivity assessment, the particles were released confirming the potential application of the composite fibers as a drug delivery system. Preliminary in vitro tests with bone marrow stromal cells were performed to investigate cell adhesion on the fabricated composite mats, the positive obtained results confirmed the suitability of the composite fibers as scaffolds for soft tissue engineering.

## 1. Introduction

The fabrication of nanocomposite fibers by using electrospinning techniques is a challenging and interesting topic for several applications, such as the development of scaffolds for tissue engineering and drug delivery [1,2,3,4,5]. The electrospinning process allows the fabrication of scaffolds with a fibrillar structure, by the application of high voltage between the conductive tip of a polymeric solution and a grounded fiber collector. The regulation of process parameters, polymeric solution parameters and environmental conditions affects the fiber morphology and properties [6].

It is possible to process many polymers and copolymers, and the selection of a suitable solvent system is fundamental to improving the electrospinning of a solution. In the last years, the spread of the “green electrospinning” concept, the reduction of the use of toxic solvents and the related increase of the use of benign solvents have been reported in the literature, for example in [7,8,9]. Limiting the use of harsh solvents is highly beneficial in terms of processing proteins, such as collagen and other sensitive biomolecules, preventing denaturation. Using benign solvents is also important to avoid the presence of toxic solvent residuals inside the mats which could limit their applications in the biomedical field, also bringing advantages in terms of lab worker safety and environmental impact [7,8,9].

Composite electrospun fibers can be obtained by the incorporation of nanoparticles inside the polymeric mats. This approach has been already widely investigated in the literature, in particular for the development of composite electrospun scaffolds for bone tissue engineering [10,11,12,13,14,15,16].

Ordered mesoporous silica particulate materials have been increasingly investigated since the first series of ordered mesoporous silica materials were developed in 1982 (known as MCM-41) [17,18]. These silica particles are characterized by ordered porosity at the nanoscale and disordered arrangement at the atomic level. After the development of MCM-41 materials, Grün et al. [19] presented the first synthesis of spherical MCM-41 nanoparticles. The most interesting features of such MCM-41 particles are the ordered mesoporous structure, hexagonally shaped pores, narrow pore size distribution (2–10 nm), and a large surface area (900–1500 m^2^·g^−1^) [17,18]. The synthesis procedure was a modification of the Stöber’s reaction [20] for the preparation of monodispersed silica spheres. Grün et al. [19] modified this procedure by adding a cationic surfactant (cetyl trimethylammonium bromide, CTAB) to the synthesis solution, providing a source of micelles. These ordered mesoporous materials have been extensively used as molecular sieves, catalyst, adsorbents and molds [17]. In 2001 [21], mesoporous silica particles were proposed for the first time as a drug delivery system. These silica-based mesoporous materials are able to incorporate a relatively high amount of drug into the mesopores. Moreover, their silanol groups can be functionalized and the pore diameter can be modulated in order to increase the drug-uptake and to better control the drug-release kinetics [22]. Due to their pure silica network, MCM-41 particles are not bioactive [23], i.e., they are not able to form hydroxyapatite layers once in contact with biological fluids (even after two months) due to the small pore size and the lower concentration of silanol groups compared to other silica particles. 

In the present work, the production of calcium-containing MCM-41 (Ca_MCM-41) particles in order to couple the drug up-take capability of the ordered mesoporous particles with the bioactivity of Ca–Si systems is reported. The obtained particles are characterized by an ordered mesoporous structure and hydroxy-carbonate apatite (HCA) formation capability after one day of immersion in simulated body fluid (SBF) solution.

A proof of concept of the incorporation of the synthesized Ca-containing MCM-41 particles in electrospun poly(epsilon-caprolactone) (PCL) fibers was performed. PCL was selected because of its biocompatibility, biodegradability, ability to be processed by electrospinning—in particular with benign solvents—and considering its FDA approval for clinical use [24,25,26]. The obtained composite nanofibers were characterized from the morphological, chemical and mechanical point of view. Even if the proposed approach of incorporating silica mesoporous nanoparticles into electrospun polymeric mats for the development of drug delivery systems has been already reported in the literature [27,28], the novelty of the present research work is represented by the synthesis optimization of the Ca_MCM-41 particles and the development of electrospinning using benign solvents aiming at the fabrication of optimized scaffolds for tissue engineering and drug delivery applications which have not been reported previously.

## 2. Materials and Methods 

### 2.1. Ca_MCM-41 Particle Synthesis

The Ca_MCM-41 particles were produced by optimizing a method available in the literature in order to obtain spherical particles with ordered mesostructured and reduced agglomeration [29]. Briefly, 0.6 g of cetyltrimethylammonium bromide (CTAB, Sigma Aldrich, Munich, Germany) were dissolved in a solution of 300 mL of deionized water and 0.2 g of sodium hydroxide (NaOH, Sigma Aldrich, Munich, Germany) under continuous stirring for up to 30 min. 3 mL tetraethyl orthosilicate (TEOS, Sigma Aldrich, Munich, Germany) was added (rate 0.25 mL·min^−1^) and the solution was stirred for 30 min. After 2.34 g of calcium nitrate (Ca(NO_3_)_2_·4H_2_O, Merck KGaA, Darmstadt, Germany) was added and the solution was stirred for 1 h 30 min. All synthesis steps were carried out at 80 °C to avoid the agglomeration of the final product. The resulting dispersion was centrifuged and washed once with deionized water and twice with ethanol. The white precipitate was then dried for 12 h at 60 °C and heat-treated at 600 °C (1 °C/min) for 6 h.

### 2.2. Degradation in Simulated Body Fluid (SBF)

Simulated body fluid (SBF) was prepared by dissolving reagent grade 8.035 g·L^−1^ NaCl, 0.355 g·L^−1^ NaHCO_3_, 0.225 g·L^−1^ KCl, 0.231 g·L^−1^ K_2_HPO_4_·(3H_2_O), 0.311 g·L^−1^ MgCl_2_ (6H_2_O), 0.292 g·L^−1^ CaCl_2_, and 0.072 g·L^−1^ Na_2_SO_4_ in deionized water and buffering at pH 7.4 at 36.5 °C with 6.118 g·L^−1^ tris(hydroxymethyl) aminomethane ((CH_2_OH)_3_CNH_2_) and 1M HCl, as previously reported by Kokubo and Takadama [30]. Ca_MCM-41 particles were immersed in SBF at a 1.5 g·L^−1^ ratio [31,32]. The specimens were kept in a polypropylene container at 37 °C in an incubator on an oscillating tray for up to seven days. The solution was not renewed and a falcon tube containing SBF as a control was also used for the entire period of the experiment, in order to control over time the stability of the testing solution. At each time point, the particles were centrifuged and washed with deionized water and dried at 60 °C overnight. The microstructural changes were investigated by means of scanning electron microscopy (SEM) equipped with an Energy Dispersive X-ray Spectrometry (EDS) detector (Auriga 0750 from ZEISS, Jena, Germany). The ordered mesoporous structure was checked by high resolution transmission electron microscopy (HR-TEM), in a Tecnai G2F30 S-Twin microscope (FEI, Eindhoven, The Netherlands) with 0.2 nm point resolution, operated at 300 kV and equipped with a HAADF Fischione detector (0.16 nm point resolution, Fischione Instrument, Pittsburgh, PA, USA). For the TEM observation, particles were homogeneously dispersed in ethanol by ultrasound and dropped on a carbon film. The pore diameter analysis of the ordered mesoporous particles was conducted on HRTEM images with ImageJ2 [33] analysis software. 

### 2.3. Electrospinning Solution and Process Parameters

Poly(epsilon-caprolactone) (PCL) (80 kDa, Sigma Aldrich, Munich, Germany) was dissolved at 15 *w*/*v*% in a mixture of formic acid (VWR International, Darmstadt, Germany) and acetic acid (VWR International, Darmstadt, Germany) in a ratio 1:1, according to the protocol reported elsewhere [10]. For the fabrication of the composites fibers, Ca containing MCM-41 particles were added to the PCL solution (same concentration of the neat sample) in a ratio of 30 wt % respect to the polymer amount. This ratio was selected according to the results of our previous investigations [10,11]. Electrospinning was performed by using a commercially available EC-CLI device (IME Technologies, Geldrop, The Netherlands). The device is equipped with a climate control which allows the setting and control of the temperature and relative humidity (RH) inside the electrospinning chamber. During the optimization of the neat PCL process, the temperature was set at 23 °C and different values of relative humidity were tested, namely 25%, 30%, 40%, 50% and 60%. The optimized value (40%), according to trial-and-error approach, was used for the fabrication of the composite mats. The device was also equipped with a gas shield module able to optimize the Taylor cone and the nitrogen flux was set at 8 mL/min. The applied voltage was 20 kV, in particular +18 kV were applied to the metallic nozzle with a diameter of 23 G and −2 kV were applied to a drum collector, rotating at 1000 rpm. The distance between the nozzle and the collector was 15 cm and both the solution and the suspension were dispensed at 1.3 mL/h. The duration of the electrospinning process was fixed for all samples at 30 min.

### 2.4. Electrospun Mats Characterization

The optimization of the RH values for the neat PCL electrospun mats was carried out by investigating the variation in fiber morphology related to the variation of the RH values. The morphology of all obtained fibers was also investigated after immersion in SBF solution using SEM after sputtering with gold (Sputter coater Q150T, Quorum Technologies, Darmstadt, Germany). Fiber average diameters and pore size were calculated by using the software ImageJ and the plugin DiameterJ [33,34] on SEM micrographs and measuring 50 fibers. ATR-FTIR spectroscopy was used for the confirmation of the incorporation of the particles inside the polymeric mats and for the bioactivity assessment. The ATR-FTIR spectra were collected in the range 4000–550 cm^−1^, with 32 scans and a resolution of 4 cm^−1^ (Nicolet 6700, Thermo Scientific, Schwerte, Germany). The mechanical properties of the electrospun mats were assessed by performing a uniaxial tensile test at room temperature, with a load cell of 50 N and a crosshead speed of 10 mm/min (K. Frank GmbH, Mannheim, Germany). The electrospun samples were cut and fixed in a paper frame, the sample measured area was 5 mm × 20 mm, as reported in a previous work [10]. The uniaxial tensile test was also performed on the composite fibers after immersion in SBF solution for one day. The acellular bioactivity test was performed by immersing the electrospun mats in SBF solution (prepared as described above) up to seven days. The ratio between the sample surface and the SBF volume was calculated according to reference [30]. Before the immersion in SBF, the electrospun mats were fixed on round scaffold supports (Scaffdex, Sigma Aldrich, Munich, Germany). Contact angle measurements were performed to assess the wettability of the electrospun mats and they were carried out by the release of 3 μL of distilled water (Krüss DSA30, Hamburg, Germany).

Preliminary cell viability and cell morphology studies on the composite mats were performed by using bone murine stromal cells ST-2 (Leibniz-Institut DSMZ—German Collection of Microorganisms and Cell Cultures GmbH, Braunschweig, Germany). Electrospun samples were cut, fixed on scaffold supports (Scaffdex, Sigma Aldrich, Munich, Germany), placed in a 24-well plate and disinfected after UV exposure for 30 min. No preconditioning was used for the electrospun mats. Before the scaffold seeding, ST-2 cells were cultured for 24 h in RPMI 1640 medium (Gibco^TM^, Thermo Fisher Scientific, Schwerte, Germany), supplemented with 10% fetal bovine serum (Lonza, Cologne, Germany) and 1% penicillin/streptomycin (Lonza, Cologne, Germany) and incubated at 37 °C with 5% CO_2_. ST-2 cells were seeded onto the electrospun mats with a density of 2×10^4^ cells/cm^2^. After 24 h, to assess cell viability, Cell Counting Kit-8 (CCK-8) assay based on WST-8 (2-(2-methoxy-4-nitrophenyl)-3-(4-nitrophenyl)-5-(2,4-disulfophenyl)-2H-tetrazolium, monosodium salt), (Sigma Aldrich, Munich, Germany) was performed in triplicate on neat PCL nanofibers and composite electrospun mats; cells seeded on the neat PCL microfibers with micrometric diameters, obtained according to [10], were used as control. Briefly, fresh RPMI 1640 medium containing 10% WST-8 was added to all the electrospun seeded samples. After sample incubation at 37 °C and 5% CO_2_ for 3 h, a microplate Elisa reader (PHOmo Elisa reader, Autobio Diagnostics Co. Ltd., Zhengzhou, China) was used to measure the absorbance at 450 nm. Cell morphology and adhesion on the composite electrospun mats was investigated by SEM analysis. Before performing the SEM analysis, the seeded samples were fixed using a fixation solution containing glutaraldehyde, paraformaldehyde, sucrose, and sodium cacodylate trihydrate (Sigma Aldrich, Munich, Germany). Subsequently, sample dehydration was obtained using a series of ethanol concentrations in deionized water. The samples were then dried in air and sputtered with gold (Sputter coater Q150T, Quorum Technologies, Darmstadt, Germany).

## 3. Results

### 3.1. Ca_MCM-41 Particles Characterization

**Figure 1:**
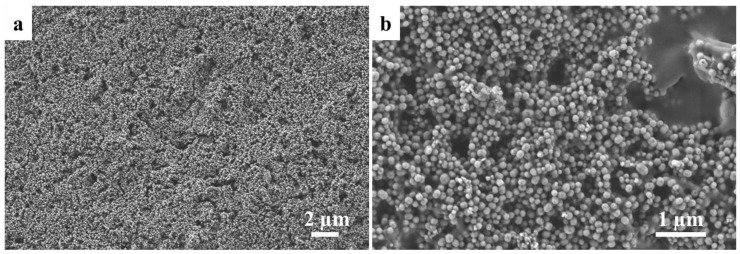
SEM micrographs of calcium-containing MCM-41 (Ca_MCM-41) particles at low magnification. (**a**) higher magnification; (**b**) show the homogeneity of particles size and shape.

The obtained material from the MCM-41 synthesis is a white fine powder characterized by low agglomeration. The morphology and the microstructure of the obtained particles were assessed by SEM and HR-TEM analysis. The Ca_MCM-41 particles were characterized by spherical shape and homogeneous size distribution, as shown in Figure 1. 

**Figure 2:**
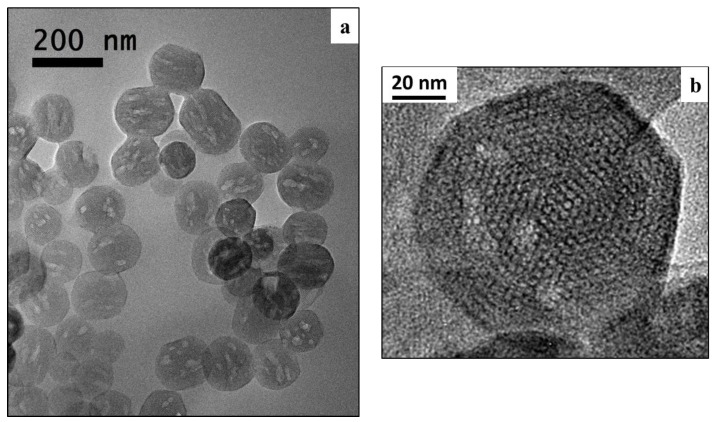
HR-TEM images of Ca_MCM-41 particles at different magnifications to evaluate the particle shape, dimension and the ordered structure of the obtained material: (**a**) overview of Ca_MCM-41 particles; (**b**) detailed view of a single particle.

The HR-TEM images, reported in Figure 2, showed the existence of a highly ordered hexagonal array and streaks, structural features confirming the formation of an ordered structure at the nanoscale.

**Figure 3:**
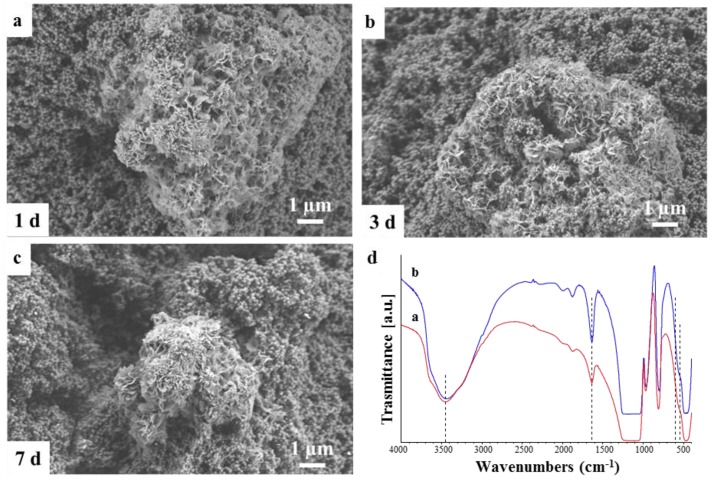
SEM micrographs and FTIR spectra of Ca_MCM-41 particles after immersion in simulated body fluid (SBF) at different time points. SEM image after 1 day (**a**), 3 days (**b**), 7 days of immersion (**c**); FTIR spectra (**d**) after 3 days (**a**) and 7 days (**b**) of immersion.

The capability of these Ca_MCM-41 particles to develop HCA was assessed in SBF solution for up to seven days of immersion. Already after one day of immersion in SBF, the formation of HCA crystals was detected by mean of SEM and reported in Figure 3. HCA crystals do not grow homogeneously on the surface of the particles, but a single HCA layer seems to develop over time, enclosing the Ca_MCM-41 particles. This formation of the HCA layer is particularly evident when comparing the SEM micrographs of the Ca_MCM-41 particles before the immersion in SBF solution (Figure 1) and the particles after immersion (Figure 3). The presence of the HCA deposition was also investigated using FTIR spectroscopy. In particular, as showed in Figure 3d, it is possible to notice the bands related to P–O asymmetric bending around 560 and 600 cm^−1^ and the bands ascribable to H_2_O molecular at 1630 and 3450 cm^−1^, correlated to the deposition of the HCA layer [35,36].

**Figure 4:**
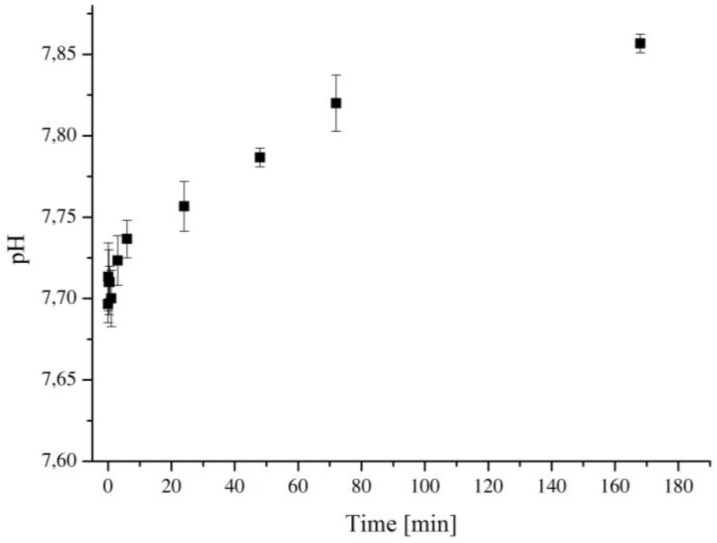
Graph of pH variation with time of the SBF solution after the immersion of Ca_MCM-41 particles.

The pH variation of the SBF solution containing the Ca_MCM-41 particles was monitored overtime and the results are shown in Figure 4. The pH of the solution increased over time, confirming indirectly that the particles released Ca^2+^ ions once in contact with SBF solution. The phenomenon of pH-increase due to the presence of Ca^2+^ ions released from silica network-based particles and its mechanisms have been already widely investigated in the literature [31,37] and the evidence of the pH increase, confirms the release of Ca^2+^ ions in the solution. Briefly, after the immersion in SBF solution, Ca_MCM-41 particles exchange Ca^2+^ with H^+^ ions from SBF solution. This exchange induces an increase in the pH of the solution and is due to the presence of mesopores, these particles showed high porosity, surface area and reactivity which accelerate the ionic exchange [37].

### 3.2. Optimization of Electrospinning Parameters

**Figure 5:**
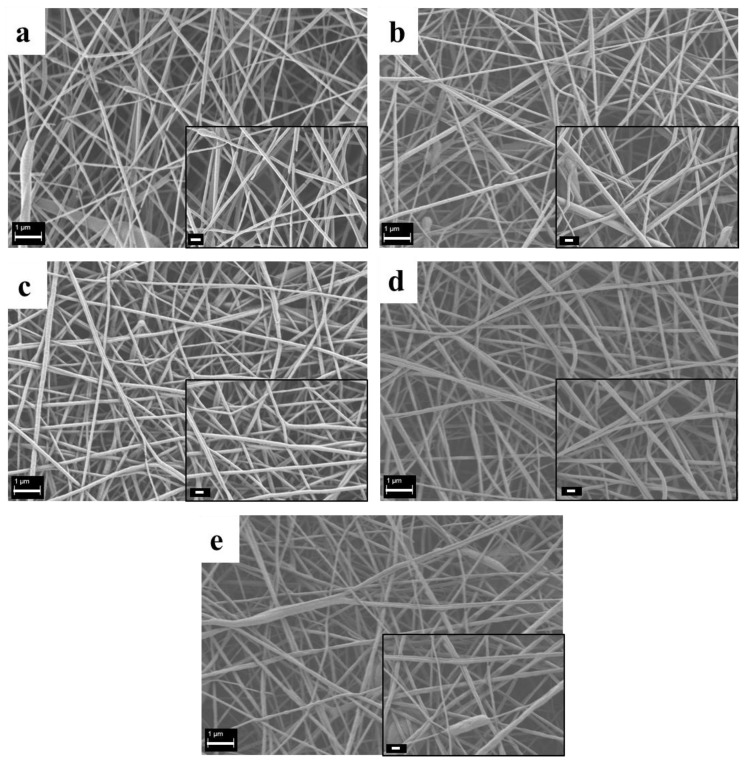
SEM analysis of neat PCL electrospun fibers obtained by setting different values of relative humidity (RH) : 25% (**a**), 30% (**b**), 40% (**c**), 50% (**d**) and 60% (**e**). Common scale bar 1 μm, higher magnification micrographs in the insets with common scale bar of 200 nm.

The optimization of the electrospinning parameters was performed on neat PCL. Different RH values were evaluated, namely 25%, 30%, 40%, 50% and 60%. SEM analysis of the obtained electrospun mats is reported in Figure 5. It was determined that in a quite wide range of RH values, between 30% and 50%, it was possible to obtain stability in terms of fiber distribution and process yield. The lowest and highest RH values, namely 25% and 60%, introduced fiber branching and non-homogeneous fiber diameter distribution, as reported in Figure 5a,e. Based on such observations, the RH value of 40% was selected as optimal and used for the fabrication of both the composite fibers and the neat PCL fibers used as a control.

**Figure 6:**
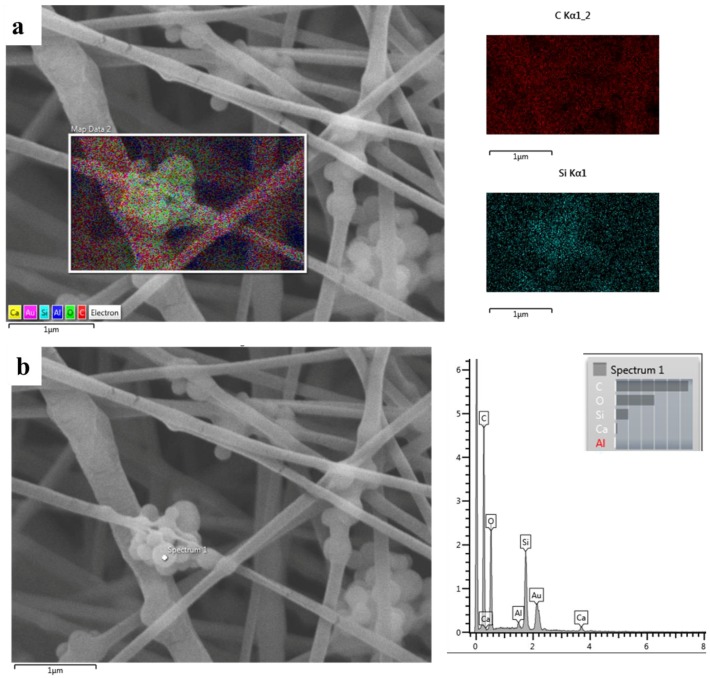
SEM/EDS analysis of the composite electrospun mats. SEM with maps and details for C (polymer component) and Si (from particles) (**a**) showing also the EDS spectrum on the particle cluster (**b**).

Composite electrospun fibers were characterized with SEM/EDS to identify particle distribution inside the polymeric matrix. The Si and Ca amounts were clearly detected in the polymeric PCL matrix, as reported by the map EDS analysis and the spectrum on the particle cluster, reported in Figure 6. The average fiber diameter of the composite mats was 257 ± 52 nm, comparable with the average fiber diameter of the neat PCL fibers that was 200 ± 40 nm, as reported in our previous study [10]. The calculated average pore size of the composite electrospun mats was around 0.6 μm^2^. This result demonstrates that the presence of the particles did not affect fiber morphology in terms of average fiber diameter and fiber diameter distribution within the indicated ranges. The contact angle measurements showed hydrophobic behavior for both neat PCL and the composite electrospun mats, in particular, the value for the neat PCL was 141 ± 3°, while the value for the composite mats was 144 ± 5°.

### 3.3. Mechanical Properties

As reported in the literature for both sample types, namely composite and neat polymeric fibers, two linear trends in the stress–strain curve could be observed. The first one is due to the load application, and the second could be ascribed to the fiber alignment before the sample fracture. The mechanical properties, such as Young’s modulus, ultimate tensile strength (UTS) and tensile strain are reported in Table 1. Comparable values were obtained for the Young’s modulus, even if the presence of surface roughness increased the distribution of the measured values, as indicated by the higher value of the standard deviation. After immersion in SBF for one day, a uniaxial tensile test was performed on the composite fibers, obtaining modulus and UTS values comparable to those of the sample before immersion.

**Figure 7:**
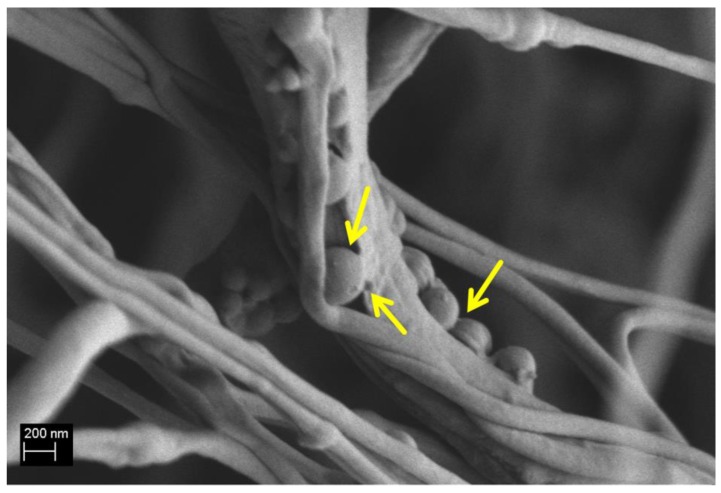
SEM micrograph of composite PCL_Ca_MCM-41 electrospun fibers after the tensile test, indicating lack of strong adhesion at the particle-PCL interface (arrows).

A reduction in UTS and tensile strain could be related to weak adhesion at the interface between Ca_MCM-41 particles and the polymeric matrix. To confirm this relation between particle adhesion and mechanical properties, SEM analysis was performed on the composite fibers after the mechanical test, as reported in Figure 7. As shown, the lack of strong adhesion between particles and the PCL matrix can be noticed, and particles have detached from the cracks in the polymeric fibers created during the tensile test.

### 3.4. Chemical Characterization

**Figure 8:**
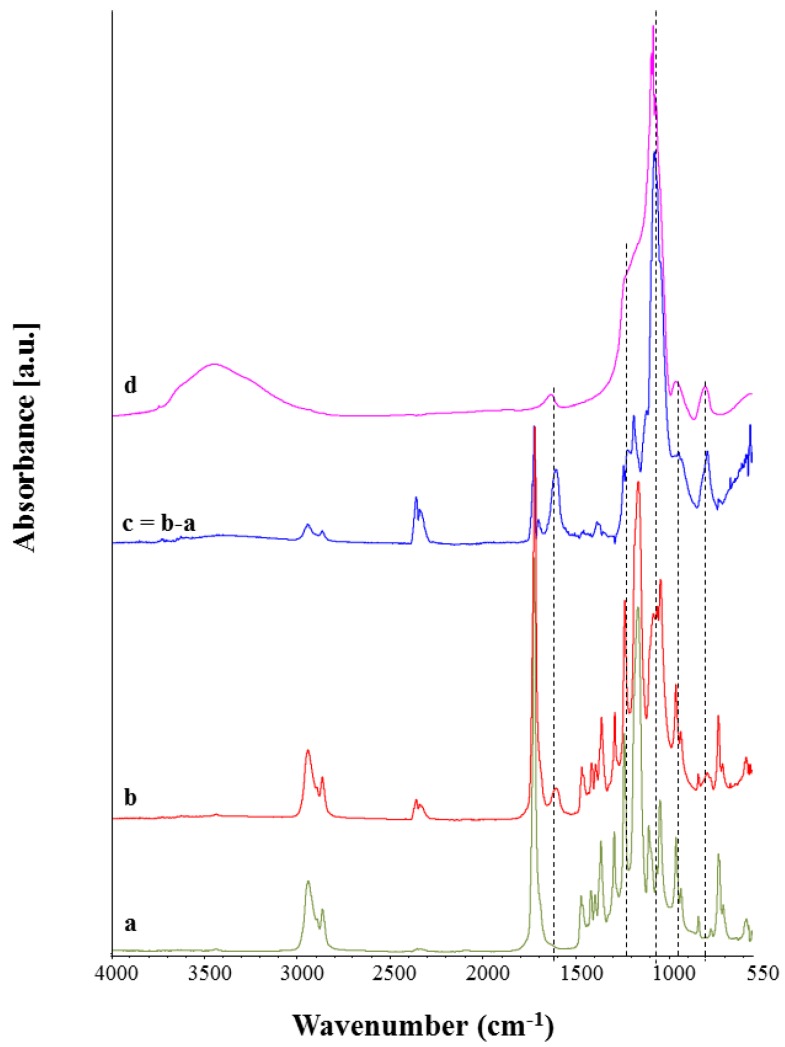
ATR-FTIR spectra of neat PCL electrospun fibers (**a**), PCL_Ca_MCM-41 composite electrospun fibers (**b**), the subtraction spectrum (neat PCL fibers subtracted from the composite fiber spectrum, (**c**) and the Ca_MCM-41 particle spectrum (**d**). Peaks of relevance are discussed in the text.

The incorporation of Ca_MCM-41 particles inside the PCL electrospun mats was investigated using ATR-FTIR analysis. In Figure 8 the FTIR spectra of the neat PCL electrospun fibers (Figure 8a), PCL_Ca_MCM-41 electrospun fibers (Figure 8b), the subtraction spectrum (neat PCL subtracted from composite spectrum, Figure 8c) and the Ca_MCM-41 spectrum (Figure 8d) are reported.

Both the spectra of the neat PCL fibers and composite fibers are dominated by the main PCL bands, like CH_2_ asymmetric and symmetric stretching vibrations around 2950 and 2865 cm^−1^, carbonyl stretching at 1720 cm^−1^, asymmetric and symmetric C–O–C stretching around 1240 cm^−1^ and 1160 cm^−1^ [11]. New bands appear in the spectrum of composite fibers (Figure 8b) and it is possible to compare them with the Ca_MCM-41 particle spectrum (Figure 8d). The presence of Ca_MCM-41 particles could be identified by observing the new bands in the composite fiber spectrum (Figure 8b). These bands are related to the presence of Si–O bonds, namely Si–O–Si stretching around 1100 cm^−1^, Si–OH stretching around 1000 cm^−1^ and Si–O stretching around 800 cm^−1^, and they are more evident in the subtraction spectrum reported in Figure 8c and indicated with dotted lines.

### 3.5. Bioactivity Assessment

**Figure 9:**
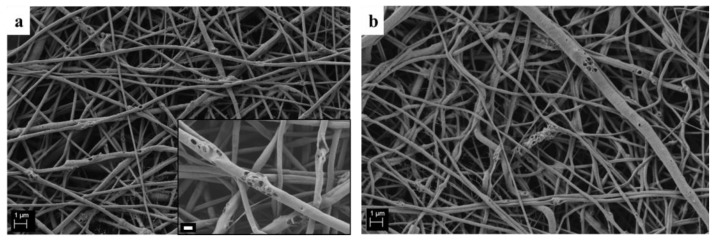
SEM micrograph of PCL_Ca_MCM-41 electrospun mats after 1 day (**a**) and 7 days (**b**) of immersion in SBF solution. Scale bar 1 μm and for the inset scale bar 200 nm.

Electrospun composite mats were immersed in SBF solution to assess the mats’ bioactivity. As reported in Figure 9, it is not possible to detect any biomineralization on the fibers, because the particles were released from the fibers already after one day of immersion in SBF solution. In fact, in Figure 9a the sample morphology after one day of immersion in SBF solution is presented, showing the presence of holes in the fibers in place of the particles. The same morphology was shown after seven days of immersion in SBF solution, as reported in Figure 9b, showing that no further fiber surface modifications occurred between one and seven days of immersion.

**Figure 10:**
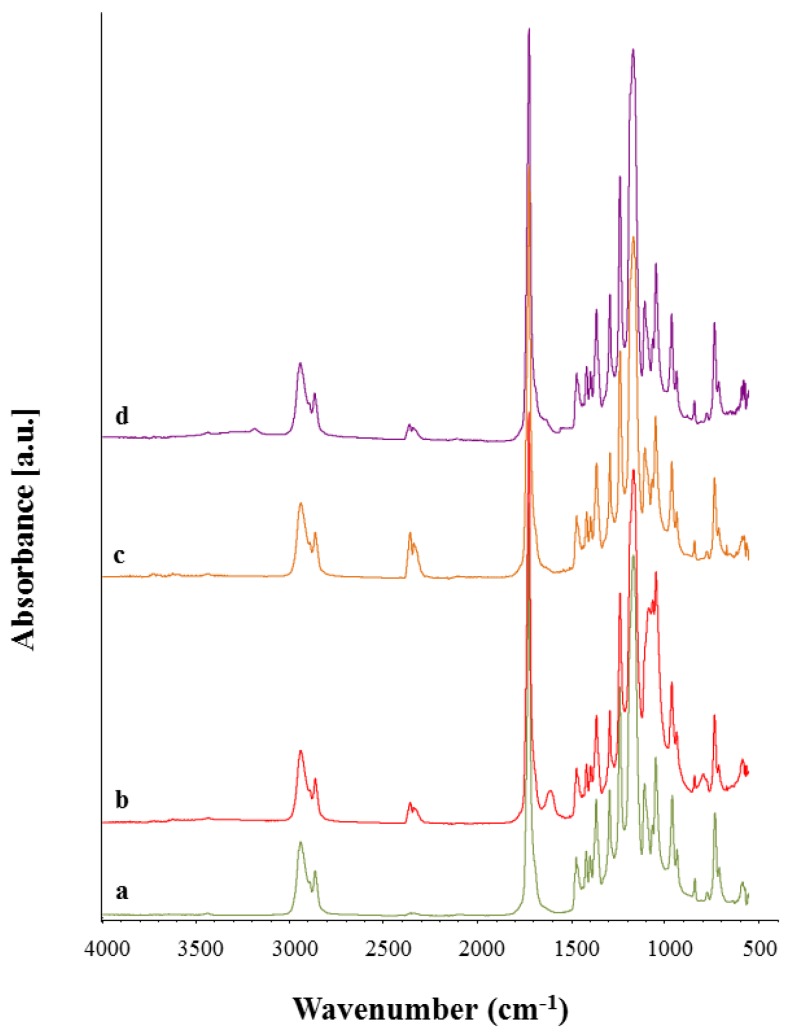
ATR-FTIR spectra of neat PCL fibers (**a**) and composite PCL_Ca_MCM-41 fibers (**b**) before immersion in SBF solution and PCL_Ca_MCM-41 fibers after immersion in SBF solution for 1 day (**c**) and 7 days (**d**).

FTIR analysis also confirmed that the presence of MCM-41 cannot be detected in the composite electrospun samples after the immersion in SBF solution. As reported in Figure 10, the bands in the range 1000–1200 cm^−1^, ascribable to Si-O bonds, that are visible in PCL_Ca-MCM-41 composite fibers before immersion (Figure 10b), are not detected in the same sample after one day of immersion in SBF solution (Figure 10c). No differences were noticed after one day or seven days of immersion in SBF solution (Figure 10c,d), because already after one day of immersion all particles were released from the composite fibers.

### 3.6. Cell-Composite Scaffold Interactions: Preliminary Tests

**Figure 11:**
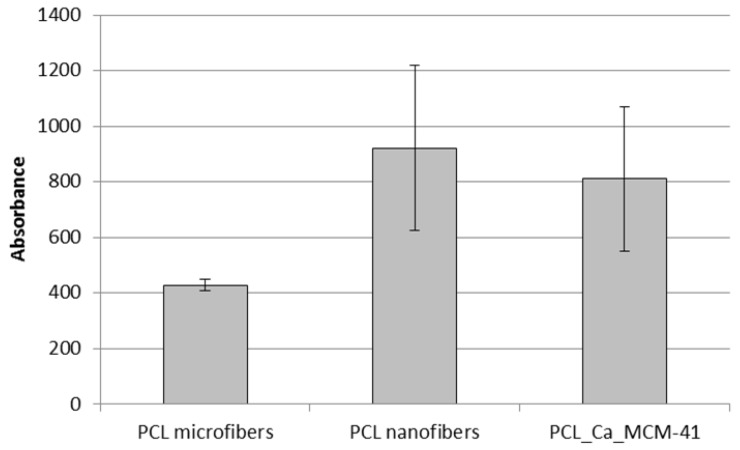
Cell viability measured by using WST-8 assay on seeded electrospun samples of neat PCL microfibers, neat PCL nanofibers and composite PCL_Ca_MCM-41 fibers.

Cell viability on the composite scaffolds was assessed by using WST-8 assay. The results showed increased cell viability for the neat PCL nanofibers and composite PCL_Ca_MCM-41 fibers with respect to the neat PCL microfibers used as a control, as reported in Figure 11. No significant differences were detected between the neat PCL nanofibers and composite PCL_Ca_MCM-41fibers, indicating that the presence of MCM-41 did not affect the cell viability on the polymeric mats.

**Figure 12:**
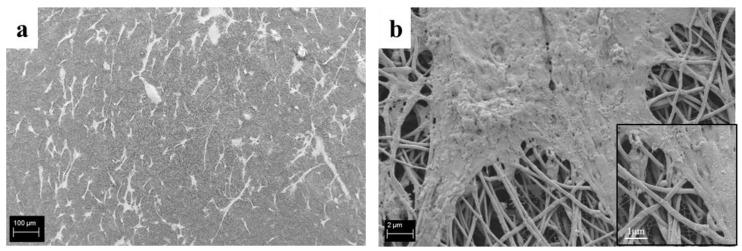
SEM micrographs of ST-2 cells seeded onto PCL_Ca_MCM-41 composite electrospun mats at different magnifications: 200× (**a**) and 10,000× (**b**).

SEM analysis performed on the seeded composite fibers to evaluate the expressed cell morphology, reported in Figure 12, showed a good adhesion of the cells on the electrospun composite mats with the typical phenotype of bone stromal cells; it is possible to observe this in the micrographs at higher magnification (Figure 12b and its inset). It is also possible to notice that the cell’s body shows a very spread morphology, following the electrospun fiber structure and orientation, and no negative influence of the fibers on cells was detected.

## 4. Discussion

The development of composite organic–inorganic fibers is attractive for several applications related to the fabrication of scaffolds for tissue engineering. 

The incorporation of mesoporous silica particles in electrospun polymeric mats has already been reported in the literature [27,28]. In particular, Song et al. [27] prepared rhodamine B (RHB)-loaded mesoporous silica particles and fluorescein (FLU)-loaded mesoporous silica particles. Both particle types were incorporated in a solution of poly(lactic-*co*-glycolic acid) (PLGA) dissolved in *N*,*N*-dimethylformamide (DMF) and tetrahydrofuran (THF), fabricating a dual-drug-loaded system. They reported a successful co-delivery system of both hydrophilic and hydrophobic drugs loaded on mesoporous silica nanoparticles. It is interesting to notice that for both drugs, the release is not related to the dissolution or detachment of the nanoparticles, since they were still visible in the SEM micrographs after the release of the drugs. Mehrasa et al. [28] also reported the successful incorporation of mesoporous silica nanoparticles in PLGA and PLGA/gelatin electrospun nanofibers. The nanoparticles were added to the polymer and blend solution and hexafluoro-2-propanol (HFP) was used as the solvent for electrospinning. They also reported an increase in the value of the Young’s modulus and the UTS related to the addition of mesoporous nanoparticles, while a reduction in the elongation was observed for the composite samples. These data are not supported by morphological analysis of fibers after tensile tests and it is not possible to compare or correlate these results with the adhesion between the particles and the polymeric matrix.

Most literature on the use of electrospinning for the incorporation of nano and/or microsized particles inside polymeric fibrous mats is related to applications to bone tissue engineering [38,39,40], but other applications for soft tissue regeneration or wound healing have also been reported [12,27,41].

In the present work, the optimization of the synthesis of calcium-containing MCM-41 particles was presented. The bioactivity of the obtained particles was confirmed. As an interesting application, these particles were dispersed in a PCL solution and processed with the electrospinning technique for potential applications as drug delivery systems and scaffolds for tissue engineering. The use of benign solvents is highlighted as this will enable the use of particles loaded with bioactive molecules for drug delivery.

The optimization of the electrospinning process parameters plays a pivotal role in the obtainment of bead-free fibrous mats, in particular when benign solvents are used. Besides the process parameters, environmental parameters also affect fiber morphology [6]. In the present work, stability in terms of fiber morphology was obtained at a wide range of RHs, between 30 and 50%.

After the optimization of the neat PCL fibers, Ca_MCM-41 particles were successfully incorporated, as shown in the SEM/EDS and FTIR analyses. The average fiber diameter was not affected by the addition of the particles, and only an increase in the standard deviation value was reported, likely due to the different rheological properties of the suspension compared to the neat PCL solution, as previously reported in the literature [10,14]. Usually, the differences in terms of solution or suspension properties require separated electrospinning optimization processes, obtaining different optimal parameters, but in the present work all the process parameters were kept constant and a comparable average fiber diameter was obtained. In literature, an increase in average fiber diameter related to the introduction of particles in the polymeric solution is usually reported, for example by Gönen et al. [14], but this increase should also relate to the particles size. In fact, in the present study the viscosity of the suspension was comparable to the neat PCL solution.

The mechanical properties of the obtained fibers were not enhanced by the addition of the particles. This trend has been already reported in the literature and it is highly likely related to the lack of strong adhesion of the particles to the polymer fibrous matrix [10,42], also documented by the SEM analysis performed on the composite fibers after the tensile tests (Figure 7). Another confirmation of the weak interaction between the inclusions and the PCL matrix was provided by the results of the uniaxial tensile tests performed after the immersion for one day in SBF, in fact the obtained comparable values showed that the mechanical properties were not modified after the release of the particles. This weak interaction between the particles and PCL matrix may be beneficial when the particles are loaded with drugs or biomolecules to facilitate the release of the molecules, without affecting the macroscopic mats fibrillary structure, preserving the mechanical function of the scaffold. In fact, after the release of the Ca_MCM-41, it is possible to observed cracks and holes on the fiber surface, but the fibers structure is preserved. Another advantage of this system is represented by the fact that the released particles are embedded in the polymer fibers. For this reason, it is possible to target the particle release in situ where the scaffold is implanted. It could be also considered that the release can be tailored by both the concentration of particles in the fibers and the extent of degradation of the fibers.

The acellular bioactivity of the composite electrospun fibers was assessed by immersion in SBF solution. Conversely from the results obtained on the Ca_MCM-41 particles, the composite PCL_Ca_MCM-41 fibers were not bioactive, in fact no deposits of HCA were observed on the fiber surface. All the particles incorporated inside the PCL fibers were already released after one day of immersion in SBF solution, leaving pores on the fiber surfaces. The release of the particles was confirmed by SEM and ATR-FTIR analyses. 

Encouraging preliminary results about cell viability and cell adhesion on the composite electrospun mats were reported. Further studies are ongoing to investigate cell migration inside the electrospun scaffolds, and cell proliferation and differentiation induced by the electrospun composite fibers.

The obtained results are relevant and broaden the field of applications of composite electrospun mats that could be used for the fabrication of scaffolds for soft tissue engineering, but also for the fabrication of multilayered scaffolds providing layers which do not require biomineralization (no bioactivity) and as complex drug delivery systems.

## 5. Conclusions

Calcium-containing MCM-41 particles were successfully synthesized and a proof of concept of their use for the fabrication of composite electrospun fibers was reported. Composite electrospun fibers were obtained after the optimization of the electrospinning parameters for both the composite sample and the neat PCL solution, used as control. Benign solvents for the electrospinning were used for the fabrication of PCL and PCL_Ca_MCM-41 composite fibers, obtaining homogeneous particle distribution inside the electrospun mats. The particle incorporation in the polymeric electrospun mats was confirmed by SEM/EDS and FTIR analysis. The bioactivity of the Ca_MCM-41 particles was not preserved, because the particles were already released from the electrospun mats after one day of immersion in SBF solution. The obtained composite fibers are promising for applications in tissue engineering involving drug delivery.

## Figures and Tables

**Table 1:** Mechanical properties of the neat PCL and PCL composite electrospun fibers.

Sample	Young’s Modulus (MPa)	UTS (MPa)	Tensile Strain (%)
Neat PCL^1^	11.0 ± 0.8	6.2 ± 0.9	115 ± 2
PCL_Ca_MCM41	13 ± 4	3.3 ± 0.9	49 ± 10
PCL_Ca_MCM-41 after SBF	10 ± 2	2.4 ± 0.4	55 ± 3

^1^ Values from reference [10].
